# Diabetic kidney disease: world wide difference of prevalence and risk factors

**Published:** 2015-10-09

**Authors:** Osama Gheith, Nashwa Farouk, Narayanan Nampoory, Medhat A Halim, Torki Al-Otaibi

**Affiliations:** ^1^Urology and Nephrology Center, Mansoura University, Egypt; ^2^Department of Nephrology, Hamed Al-Essa Organ Transplant Center, Kuwait; ^3^Faculty of Nursing, Mansoura University, Mansoura, Egypt; ^4^Dasman Diabetes institute, Kuwait

**Keywords:** Diabetic kidney disease, End-stage kidney disease, Glomerular filtration rate, Type 2 diabetes, Microalbuminuria, Chronic kidney disease

## Abstract

Diabetic kidney disease – which is defined by elevated urine albumin excretion or reduced glomerular filtration rate (GFR) or both – is a serious complication that occurs in 20% to 40% of all diabetics. In this review we try to highlight the prevalence of diabetic nephropathy which is not uncommon complication of diabetes all over the world. The prevalence of diabetes worldwide has extended epidemic magnitudes and is expected to affect more than 350 million people by the year 2035. There is marked racial/ethnic besides international difference in the epidemiology of diabetic kidney disease which could be explained by the differences in economic viability and governmental infrastructures. Approximately one-third of diabetic patients showed microalbuminuria after 15 years of disease duration and less than half develop real nephropathy. Diabetic kidney disease (DKD) is more frequent in African-Americans, Asian-Americans, and Native Americans. Progressive kidney disease is more frequent in Caucasians patients with type 1 than type 2 diabetes mellitus (DM), although its overall prevalence in the diabetic population is higher in patients with type 2 DM while this type of DM is more prevalent. Hyperglycemia is well known risk factor for in addition to other risk factors like male sex, obesity, hypertension, chronic inflammation, resistance to insulin, hypovitaminosis D, and dyslipidemia and some genetic loci and polymorphisms in specific genes. Management of its modifiable risk factors might help in reducing its incidence in the nearby future.

**Implication for health policy/practice/research/medical education:**

Diabetic kidney disease is not uncommon complication of diabetes (type 1 and 2) all over the world and among geriatric population. Early and tight control of diabetes is the corner stone for the management of diabetic kidney disease (DKD). More epidemiological studies are needed to evaluate the size of the problem especially in high risk group.

## Introduction

Diabetic kidney disease (DKD) is a thoughtful complication that take place in 20% to 40% of all diabetics. In the Western world, diabetic kidney disease is the primary single cause of end-stage kidney disease (ESKD) (1). Both type 1 and type 2 diabetes can lead to nephropathy, but in type 2 diabetes, a smaller proportion of patients progress to ESKD. Because of higher prevalence of type 2 diabetes, these patients represent more than half of diabetics on hemodialysis(2). The incidence of DKD as a cause of ESKD is increasing each year (1). For clinical care and epidemiological studies, DKD is characterized by raised urine albumin excretion or reduced glomerular filtration rate (GFR), or both (3).

The prevalence of diabetes around the world has reached epidemic proportions. While diabetes is already estimated to affect more than 8% of the global population (nearly more than 350 million people), this is predictable to grow to over 550 million people by the year 2035 (4). It has been estimated that more than 40% of people with diabetes will develop chronic kidney disease (CKD),(5) including a significant number who will develop ESKD requiring renal replacement therapies (dialysis and or transplantation).

## Materials and Methods

For this review, we used a variety of sources by searching through PubMed, EMBASE, Scopus and directory of open access journals (DOAJ). The search was performed by using combinations of the following key words and or their equivalents; diabetic kidney disease, end-stage kidney disease, glomerular filtration rate, type 2 diabetes, chronic kidney disease and microalbuminuria.

## Results

Diabetic kidney disease is uncommon if diabetes is less than one decade duration. The highest incidence rates of 3% per year are on average seen 10 to 20 years after diabetes onset, after which the rate of nephropathy tapers off. It is worth wise to say that a diabetic patient for 20 to 25 years without clinical signs of DKD has low chance to develop such complication (only a 1% year)(6). There is marked racial/ethnic and international difference in the epidemiology of DKD (7,8). Native Americans, Hispanics and African-Americans have a much greater risk of developing ESKD than non-Hispanic whites with type 2 diabetes (7). Based on 2002 US data, diabetes is the cause of renal disease in 44% to 45% of incident ESKD cases, making the US rate one of the highest worldwide (8).Internationally, considerable variability among countries, with percentages fluctuating from nine percent in Russia to forty nine percent in Malaysia. This discrepancy could be explained by the differences in economic viability and governmental infrastructures (9).

## Stages of diabetic nephropathy

DKD is a chronic complication of both type 1 diabetes mellitus (DM) (beta cell damage, absolute lack of insulin) and type 2 DM (insulin resistance and/or decreased secretion of insulin) (10).There are five stages in the development of diabetic nephropathy. Stage I, GFR is either normal or increased; lasts around 5 years from the onset of the diabetes. The size of the kidneys is increased by nearly 20% and renal plasma flow is increased by 10%-15%, but without albuminuria or hypertension. Stage II, starts more or less 2 years after the onset of the disease with thickening of basement membrane and mesangial proliferation with normalization of GFR but without clinical signs of the disease. Many patients continue in this stage for life. However, stage III, represents the first clinically detectable sign of glomerular damage and microalbuminuria (albumin 30-300 mg/day). It usually occurs 5 to 10 years after the onset of the disease with or without hypertension. Approximately 40% of patients reach this stage. Stage IV, is the** s**tage of CKD with irreversible proteinuria (>300 mg/day), decreased GFR below 60 mL/min/1.73 m^2^, and sustained hypertension. Stage V**,** is defined when ESKD with GFR <15 mL/min/1.73 m^2^ is detected. Nearly 50% of patients will need renal replacement therapy in the form of peritoneal dialysis, hemodialysis or kidney transplantation (11).In the early stages of diabetic nephropathy, nephromegaly and changed Doppler indicators may be the early morphological signs of renal damage, however proteinuria and GFR are the best indicators of the degree of the damage (12).

The predictive value of microalbuminuria for the progression of kidney damage in patients with type 1 or 2 DM was confirmed in the early 1980s (13). Almost 20% to 30% of the patients progress to microalbuminuria after 15 years of disease duration and less than half develop real nephropathy (14). The European Diabetes (EURODIAB) Prospective Complications Study Group (15) and 18-year Danish study (16)reported overall occurrence of microalbuminuria (after 7.3 years) in patients with type 1 and 2 DM as 12.6% and 33%, respectively. Conferring to the United Kingdom Prospective Diabetes Study (UKPDS), the incidence of microalbuminuria in patients with type 2 DM in the United Kingdom is 2% per year and the prevalence is 25% ten years after the diagnosis(8). Proteinuria develops more frequent in patients with type 1 diabetes (15%-40%), usually after 15-20 years of DM duration,(17)but in patients with type 2 DM, the prevalence varies between 5% and 20% (4).

## Risk factors for diabetic kidney disease in type 1 diabetes

Hyperglycemia is well known risk factor for DKD and it recognized that intensive glucose control reduces the risk of DKD(4). Specifically, during the Diabetes Control and Complications Study (DCCT), nearly normalization of blood sugar decreased the risks of incident microalbuminuria and macroalbuminuria by 39% (95% CI 21%-52%) and 54% (95% CI 29%-74%), respectively, compared with conventional therapy. Even with long term follow up in observational Epidemiology of Diabetes Interventions and Complications (EDIC) Study, formerly assigned patients to DCCT intensive therapy study continued to experience lower rates of incident microalbuminuria and macroalbuminuria with risk reductions of 45% (95% CI 26%-59%) and 61% (95% CI 41%-74%), respectively (18). Beneficial effects of intensive therapy on the worsening of GFR have become evident during long-term combined DCCT/EDIC follow-up, with a risk reduction of 50% (95% CI 18%-69%). Other risk factors for DKD in diabetics include male sex, obesity, hypertension, inflammation, resistance to insulin, hypovitaminosis D, and dyslipidaemia (4,8,19). Moreover, a hereditary component to DKD has long been recognized as some genetic loci and polymorphisms in specific genes have been associated with DKD.

## Diabetic kidney disease in type 1 diabetes

During the last century, landmark studies of type 1 diabetes considered the natural history of DKD as progressive increase of urine albumin excretion followed by GFR loss and the development of ESKD. Microalbuminuria was defined as albumin excretion rate (AER) 30 to 299 mg/24 h “incipient nephropathy,” progressed steadily to macroalbuminuria with AER ≥300 mg/24 h “diabetic nephropathy.” Microalbuminuric patients commonly noted to have higher GFR “hyperfiltration,” while macroalbuminuric patients showed rapid GFR loss leading steadily to ESKD. Frequent exceptions had been observed. Specifically, albuminuria has been observed to revert, while GFR loss has been observed without albuminuria and is not always progressive. Therefore, albuminuria and impaired GFR are not necessary complementary, overlapping manifestations of DKD(4).

## Incidence of diabetic kidney disease in type 1 diabetes

Nearly half of patients with type 1 DM develop DKD over the course of their lifetime. However, albuminuria and reduced GFR both are infrequent during the first 10 years after type 1 diabetes diagnosis(4). In more recent studies, the lifetime increasing incidence of macroalbuminuria has been defined as 15%-25%, and the cumulative incidence of microalbuminuria has been reported as 25%-40% (19). In early studies, up to 35% of participants developed end stage kidney disease. In Finland and in the Pittsburgh Epidemiology of Diabetes Cohort (Pittsburgh, PA, USA), the long-term cumulative incidence of ESKD has dropped to less than 10%, though the rate of ESKD has remained higher in the Joslin type 1 diabetes cohort (Boston, MA, USA) (20).

## Progression of diabetic kidney disease in type 1 diabetes

The progression of DKD in type 1 diabetes is unpredictable. In the Joslin type 1 diabetes cohort, 29% of participants with microalbuminuria showed reduced GFR within 12 years’ average follow-up. EURODIAB type 1 diabetes study reported that 14% of microalbuminuric patients developed macroalbuminuria above 7.3 years’ follow-up. Steno type 1 diabetes cohort showed that 34% of participants with microalbuminuria went on to develop macroalbuminuria over 7.5 years’ average follow-up (17).In the DCCT/EDIC cohort, participants who had incident microalbuminuria, the 10-year cumulative incidence of macroalbuminuria was 28% (19).

In the DCCT/EDIC cohort, patients with macroalbuminuria lost GFR at a mean rate of 5.7% per year, and the 10-year cumulative incidence of impaired GFR was 32%.While in patients with microalbuminuria, mean rate of estimated GFR loss was 1.2% per year, and the 10-year cumulative incidence of diminished GFR was 15%. Interestingly, in the Joslin type 1 diabetes cohort, “early renal function decline” developed in nearly one third of microalbuminuric participants and also occurred occasionally in persistent normoalbuminuric participants with (AER <30 mg/24 hour). Such findings suggest that albuminuria and GFR loss are interrelated but are not essentially indicative of a single, homogenous underlying disease process(8).

## Regression of kidney disease in type 1 diabetes

Microalbuminuria commonly regresses to normoalbuminuria as reported in the Joslin type 1 diabetes cohort. They showed that 58% of patients with persistent microalbuminuria regressed to persistent normoalbuminuria over the next 6 years, frequently without inhibitors of the renin-angiotensin-aldosterone system (RAAS) (17).Similar results were observed in the DCCT/EDIC (8,17). Therefore, better control of diabetes, hypertension and lipids were associated with a greater likelihood of microalbuminuria regression. Of DCCT/EDIC participants who developed macroalbuminuria, more than half regressed to sustained microalbuminuria or even normoalbuminuria within 10 years, but many were managed with RAAS inhibitors (8).** **Furthermore, improvement of macroalbuminuria was associated with an 89% lower risk of progressing to reduced GFR. In the same direction, longitudinal studies of pancreas transplantation demonstrate that the pathological lesions of diabetic glomerulopathy can regress with euglycemia^.^(20).

## Diabetic kidney disease in type 2 diabetes

The incidence of DKD and rates of its development are less clear in type 2 compared with type 1 diabetes, mainly due to the highly variable age of onset, complexity of defining the exact time of diabetes onset, and the relative scarcity of long-term type 2 diabetes cohorts. Therefore, two of the best characterized type 2 diabetes cohorts are the UKPDS and the Pima Indian population. The UKPDS enrolled more than 5000 participants with new-onset type 2 diabetes and after a median 15 years of follow-up, they found that microalbuminuria (defined as persistent albuminuria ≥50 mg/L) occurred in 38% of participants, and reduced GFR (defined as persistent eGFR ≤60 mL/min/1.73m^2^) occurred in 29% of participants (21). Among Pima Indians, for whom the onset and duration of diabetes are more surely determined due to systematic diabetes screening, the cumulative incidence of macro-proteinuria (≥1 gram per gram creatinine) was 50% at 20 years’ duration, prior to widespread use of RAAS inhibitors. Despite the stable high rate of macro-proteinuria in the Pima population over time, the incidence of ESKD has declined (22).

The prevalence of DKD in most type 2 diabetics - at any point in time - is approximately 30%-50%, and this was reported among US diabetic adults (>90% type 2). This prevalence was ranging between 25% in patients younger than 65 years old to nearly 50% with age older than 65 years (22).At younger ages, microalbuminuria predominates while in older age reduced GFR is increasingly prevalent among cases with DKD. This finding could be explained by the trend in using medications that reduce albuminuria, such as glucose-lowering medications and RAS inhibitors.

Nevertheless, the phenotype of non-proteinuric DKD has been increasingly recognized in type 2 diabetes. In population-based studies of diabetes in the United States and Australia, 36%-55% of individuals with reduced GFR without concurrent albuminuria. Frequently, non-proteinuric DKD was observed in the absence of diabetic retinopathy, signifying other mechanisms than diabetic glomerulopathy. In the UKPDS, female sex, older age, and resistance to insulin were risk factors for reduced GFR but not microalbuminuria, while male sex, obesity, hyperglycemia, and hyperlipidemia were risk factors for microalbuminuria but not reduced GFR (20). Higher blood pressure was a risk factor for both reduced GFR and microalbuminuria.

## Progression of diabetic kidney disease in type 2 diabetes

The progression and regression of established DKD is highly variable in type 2 diabetes. In the UKPDS, evolution from microalbuminuria to macroalbuminuria occurred at a rate of 2.8% per year, and change over from macroalbuminuria to renal dysfunction or ESKD occurred at a rate of 2.3% per year.Similar to what happened with type 1 diabetes; loss of GFR can occur at any level of albuminuria but tends to be more rapid with greater urine albumin excretion. At diagnosis of type 2 diabetes, 7.3% of patients had microalbuminuria and it got worse by time to 17.3% at 5 years, 24.9% at 10 and 28.0% at 15 years(23).

## Health consequences of diabetic kidney disease

The high mortality risk observed among people with both types 1 and 2 diabetes is largely confined to those with evidence of DKD because it is associated with a number of interrelated cardiovascular diseases, including micro, and macroangiopathies.

## Diabetic kidney disease in different countries

Diabetic kidney disease is more frequent in African-Americans, Asian-Americans, and Native Americans (24).Progressive kidney disease is more frequent in Caucasian patients with type 1 than type 2 DM, though its higher prevalence in type 2 diabetic patients because this type of DM is more prevalent (25). The occurrence of diabetic kidney disease in Pima Indians is very interesting, indeed. Craig et al reported that around 50% of Pima Indians with type 2 DM developed nephropathy after 20 years of the disease, and 15% of them had reached ESKD (26).In the United States, the occurrence of DKD in patients starting kidney replacement therapy doubled in the late ninety (24). Fortunately, the trend has been reducing, mostly because of better prevention and earlier diagnosis and treatment of DM (27).

In the United States, 25.6 million adults (11.3%) aged 20 years and more had diabetes in 2011, with prevalence increasing in older age groups (26.9% of people aged ≥65 years). However, nearly 3% of newly diagnosed patients with type 2 DM have overt nephropathy. Among people with diabetes, the prevalence of DKD remained stable(3).Approximately 44% of new patients who are starting dialysis in the United States are diabetics. Therefore, early diagnosis of diabetes and early intervention are critical in preventing development of renal failure seen in many type 1 and a significant percentage of type 2 diabetics. The prevalence of diabetes – especially type 2 – is greater in certain races and ethnic groups, affecting approximately 13% of African Americans, 9.5% of Hispanics, and 15% of Native Americans (28,29).Nearly 20% to 30% of all diabetics will progress to evident nephropathy, although a greater percentage of type 1 patients progress to ESKD.

Epidemiologic differences occur among European countries mainly Germany. The proportion of patients admitted for renal replacement therapy is higher than reported from the United States. In Heidelberg (southwest of Germany), nearly 60% of patients who were starting renal replacement therapy in 1995 had diabetes with the majority (90%) of type 2 DM. An increase in ESKD secondary to type 2 DM has been noted even in countries known to have low incidences of type 2 DM, such as Denmark and Australia. However, the exact Asian incidence and prevalence are not readily available(3).

Pavkov et al (30)reported that DN affects males and females equally, and it rarely developed before 10 years’ duration of type 1 DM. The role of age in the development of DKD is unclear despite that the mean age of patients who reach ESKD is about 60 years and the incidence of DKD is higher among elderly individuals who have had type 2 diabetes for a longer generation. In Pima Indians with type 2 diabetes, the earlier the onset the disease at a younger age is the greater the risk of evolution to ESKD. Moreover, the incidence and severity of diabetic kidney disease is 3 to 6-fold higher in blacks than in whites. Similarly, DN is more common among Mexican Americans and Pima Indians with type 2 DM. This suggests that socioeconomic factors, such as diet, poor hyperglycemia control, hypertension, and obesity, have a main role in the progression of diabetic kidney disease. Familial clustering may be one of the important factors in these populations.

Bhalla et al(31) reported that some racial/ethnic minorities with type 2 diabetes were more probable to have proteinuric DKD rather than non-proteinuric DKD.

DN has become an important clinical and public health challenge as reported by de Boer and colleagues(3)who estimated the disease burden in the United States adult people older than 20 years through analysis of data from the National Health and Nutrition Examination Survey (NHNES).

Parving et al (32) reported the prevalence of micro-/macroalbuminuria in a cross sectional study among 32 208 type 2 diabetes patients’ from 33 countries as 38.8% and 9.8%, respectively. Asian and Hispanic patients had the highest prevalence of microalbuminuria (43.2% and 43.8%) and macroalbuminuria (12.3% and 10.3%) while Caucasians had the lowest microalbuminuria (33.3%) and macroalbuminuria (7.6%). Twenty-two percent of patients had compromised renal function (GFR <60 ml/min/1.73 m^2^). Unnikrishnan et al(33) reported that the prevalence of overt nephropathy and microalbuminuria was 2.2% and 26.9%, correspondingly, among urban Asian Indians with type 2 diabetes. Among 8897 Japanese type 2 diabetes subjects from 29 medical clinics or general/university-affiliated hospitals from different areas, the prevalence of microalbuminuria and decreased GFR (<60 ml/min per 1.73 m^2^) was 31.6% and 10.5%, respectively(34).

In the US population, the pathways study-across-sectional analysis among 2969 primary care diabetics of a large local health maintenance organization observed the racial/ethnic differences in early DN despite comparable access to diabetes care. Among non-hypertensives microalbuminuria was 2 fold larger (odds ratio [OR]: 2.01; 95% CI: 1.14–3.53) and macroalbuminuria was 3 fold larger (OR: 3.17; 95% CI: 1.09–9.26) for Asians as compared with whites. Among hypertensive patients, adjusted odds of microalbuminuria were greater for Hispanics (OR: 3.82; 95% CI: 1.16–12.57) compared to whites, while adjusted odds of macroalbuminuria were 3 fold greater for blacks (OR: 3.32; 95% CI: 1.26–8.76) than for whites (35).

What is new is the recent unreasonable rise in the prevalence of metabolic syndrome(36) and of type 2 diabetes(37)worldwide, which is extremely pronounced in Asian countries,(37) especially in India known to be the “capital of diabetes world” (38-41). Indian diabetics have a propensity to have insulin resistance, greater waist circumference despite lower body mass index as well as lower adiponectin and higher inflammatory markers (41). The prevalence of overt diabetes is predominantly high in Indian elderly (42) in addition to prediabetes and overt diabetes in the young (40-43), and consequently diabetic kidney disease especially in the rural populations of India (44-48). The estimated overall incidence rate of CKD and ESKD in India is currently 800 per million population and 150–200 per million population, respectively (48).

Of great interest is the fact that the risks of impaired fasting glucose and of impaired glucose tolerance are markedly higher in citizens of a South-East Asian origin compared with the local populations of European origin (49). Furthermore, the prevalence of any type of CKD and its rate of progression, specially DKD, is significantly higher in citizens of Asian origin, as observed both in the United Kingdom (50) and in Canada (51) presumably the result of different genetics and/or lifestyle and lack of awareness of kidney complications of diabetes (52).

As many as six Arabic speaking countries - Kuwait, Lebanon, Qatar, Saudi Arabia, Bahrain, and United Arab Emirates (UAE), are among the world’s leaders in terms of type II diabetes prevalence. Rapid economic growth brings with it great opportunities for improvements in infrastructure (e.g., health care and education), in addition to the burden of greater dependence on modernization, a proliferation of Western-style fast food, access to cheap migrant manual labors, and greater occasions for sedentary lifestyles, especially in the young (53).

In a cross-sectional study from Egypt, 42% of diabetics had nephropathy (54), in Jordan, 33% of diabetics at a national diabetes center had nephropathy (55)and at a diabetic clinic in Libya 25% of patients had nephropathy (56).

## Geriatrics and diabetic kidney disease

Increase in the prevalence of DKD also derives directly from the growth in its prevalence among individuals older than 65 years. Individuals older than 65 years are unduly affected by diabetes and related end-stage renal disease. According to data from the NHNES, the prevalence of diabetes was 26.9% among people aged ≥65 years (57,58). The prevalence of diabetic kidney disease was increased from 7.1% in 1988–1994 to 8.6% in 1999–2004 and 10.7% in 2005–2008 among individuals aged 65 years and older (3,59). Recent data also revealed that the adjusted point prevalence rates per million population of reported diabetes-related ESKD for individuals aged 60–69 and ≥70 years were 410.3 and 475.7 in whites and 1439.9 and 1471.5 in African (60,61).

One of the challenges of managing the elderly with DKD is that they may develop more complications, especially microangiopathies and retinopathy. In its 2011 National Diabetes Fact Sheet, the Centers for Disease Control (CDC) reported that in 2004, heart disease and prior stroke were, respectively, noted on 68% and 16% of diabetes-related death certificates among people aged 65 years or older (57). Moreover, the CDC indicated that, 27% of adults in 2005 with diabetes who were 75 years or older reported some degree of visual impairment compared with 15% of diabetics who were between 18 to 44 years of age (57). Individuals aged 65 years or older account for 55% of diabetic subjects who had non-traumatic lower extremity amputations (62). Caring for elderly DKD patients imposes a huge financial load on governments and family members. For example, the American Diabetes Association (ADA) indicated that the entire estimated cost of diabetes in 2007 was $174 billion, including $58 billion to treat diabetic chronic complications (63).

DKD in the elderly is mainly due to diabetes type 2 and its distribution is irregular among different racial groups. American-Indians, African-Americans, and Mexican-Americans have a greater incidence than Caucasians by as much as three to one depending on the minority cohort selected for comparison (61).

Nearly all studies demonstrating beneficial effects of metabolic and blood pressure controls on DKD have been implemented in younger aged cohorts. Importantly, the management of DKD in older people is frequently based on extrapolations of data gathered in selected and motivated younger people. Moreover, people older than 70 years have been almost excluded in trials supporting major US practice guidelines for the use of angiotensin-converting enzyme inhibitors (ACEI) and angiotensin receptor blockers (ARB) in CKD. In managing DKD in the elderly, physicians should keep in mind several key points: first; elderly diabetics constitute a different group expressing various clinical and functional situations. Second; the American Geriatric Society Panel on Improving Care for Elders with Diabetes (PICED) recommends that treatment of elderly patients with diabetes focus on specific problems and priorities(64).Third; the American Geriatric Society has also introduced the concept of time prospect for the benefits of certain treatments. Better glycemic control may take as long as 8 years to have positive results on microangiopathies. Benefits of good hypertension and dyslipidemia control may not be noticeable before 2 or 3 years (65). Forth; many elderly patients with diabetes are fragile and are also at greater risk for developing several common syndromes, such as intellectual impairment, depression, urinary incontinence, injurious falls, and persistent pain. The Assessing Care of Vulnerable Elders (ACOVE) project describes a frail elderly patient as a vulnerable elder person than 65 years and is at increased risk of death or functional decline within 2 years (65). Fifth; in consequence, renoprotection in elderly population should be tailored based on patients’ autonomy, degree of frailty, life expectancy, co-morbidity index, and the stage of DKD and finally sixth; elderly diabetic patients may be susceptible to nephrotoxic agents as radiocontrast material; particular attention must be taken in preventing and monitoring radiocontrast induced nephropathy.

## Conclusion

Diabetic kidney disease is not uncommon complication of diabetes (type 1 and 2) all over the world and among geriatric population. Management of its modifiable risk factors might help in reducing its incidence in the near future.

## Authors’ contribution

OG: Conception, design, drafting the article, critical revising of important intellectual content, final approval of manuscript. NF: share in conception, design, drafting the article, critical revising of important intellectual content, and final approval of manuscript. NN: Share in critical revising of important intellectual content, final approval of manuscript. MAH: Share in critical revising of important intellectual content, final approval of manuscript. TAO: Share in critical revising of important intellectual content, final approval of manuscript.

## Conflicts of interest

The authors declared no competing interests.

## Ethical considerations

Ethical issues (including plagiarism, data fabrication, double publication) have been completely observed by the authors.

## Funding/Support

None.
